# Supplementary material for: Dominance of the scleractinian coral Alveopora japonica in the barren subtidal hard bottom of high-latitude Jeju Island off the south coast of Korea assessed by high-resolution underwater images
Source: PLoS One. 2022 Nov 4;17(11):e0275244. doi: 10.1371/journal.pone.0275244 (PMC9635743; doi:10.1371/journal.pone.0275244)

Using Image Analysis Software

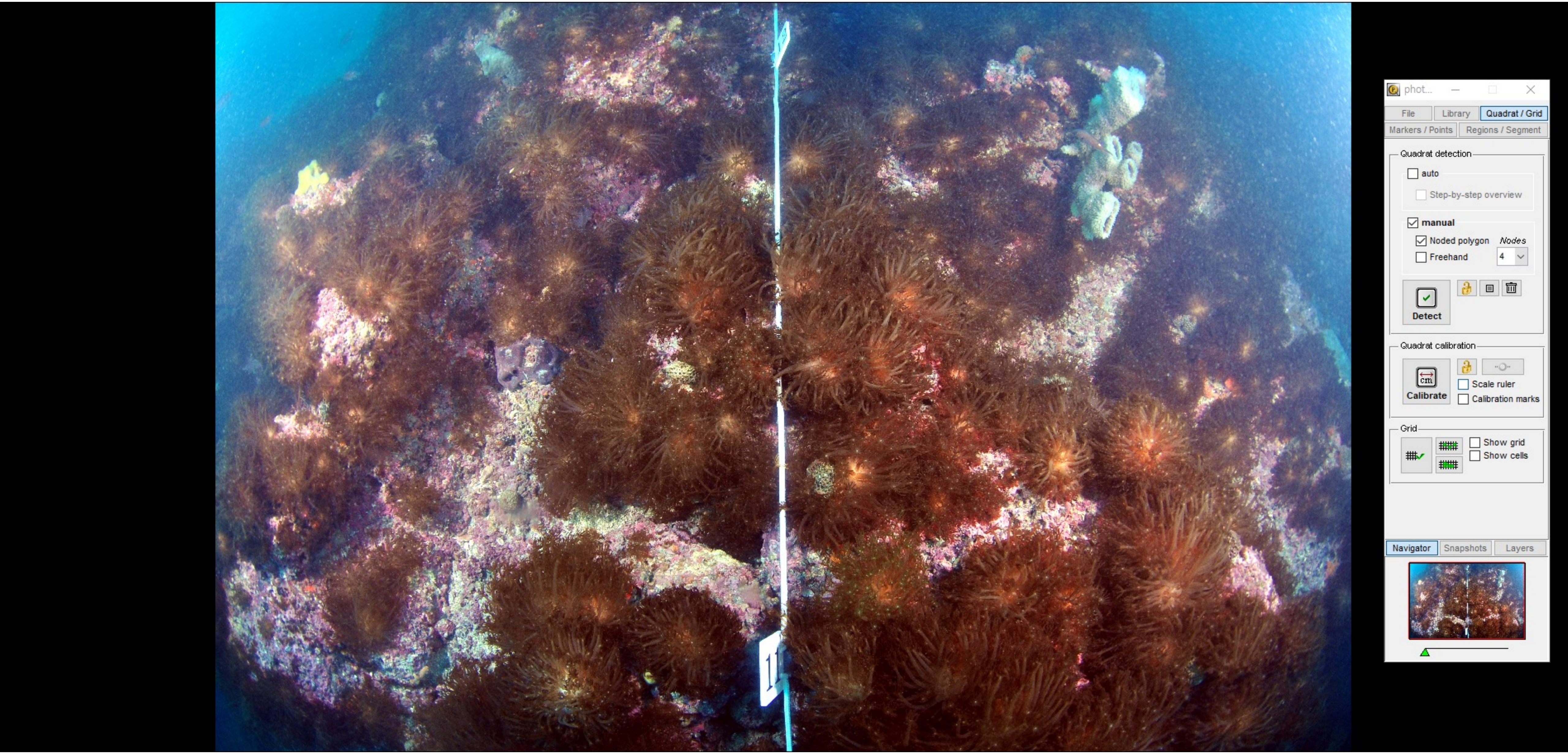

1. Image calibration (Scale on the photo)

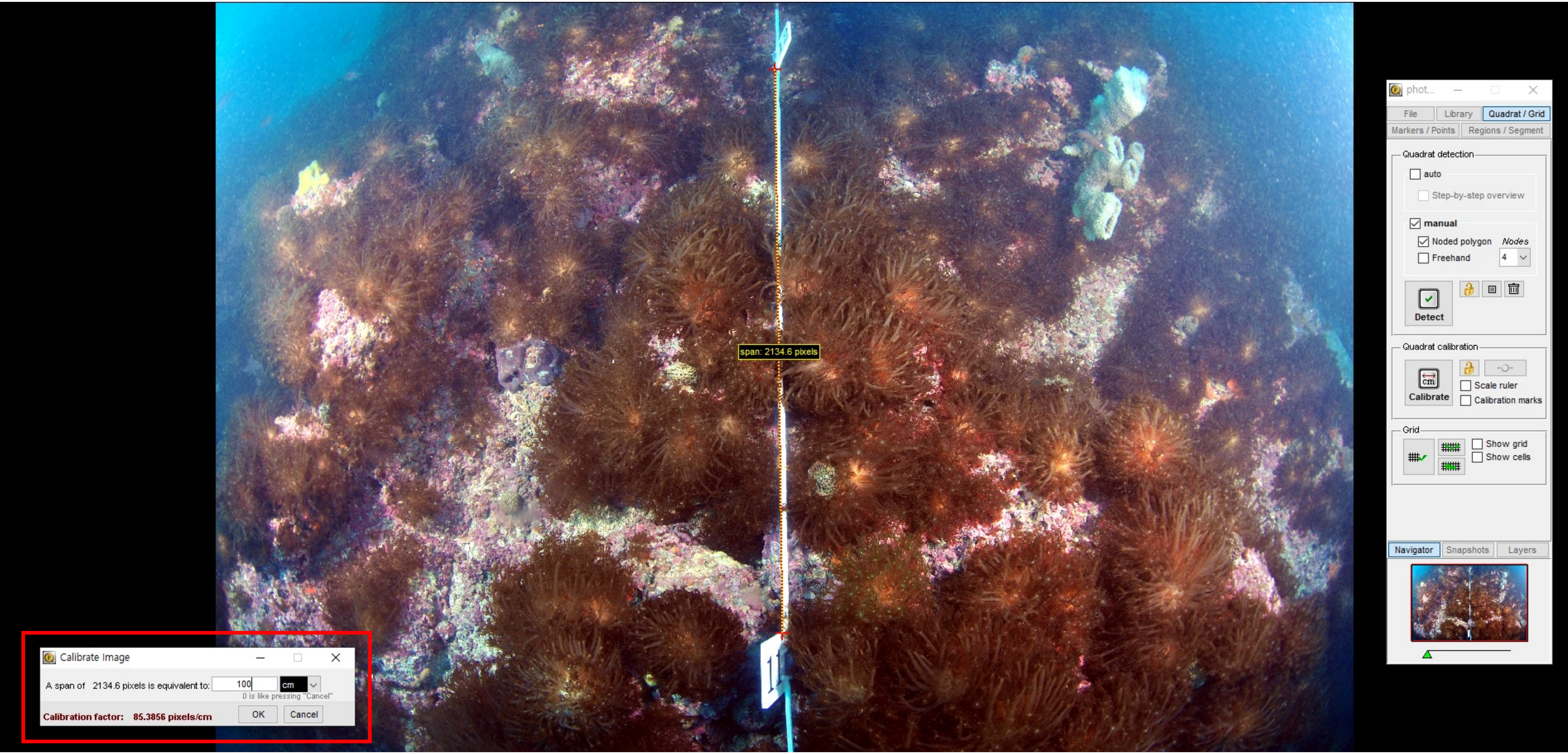

2. Measure tools (Distance, Angle)

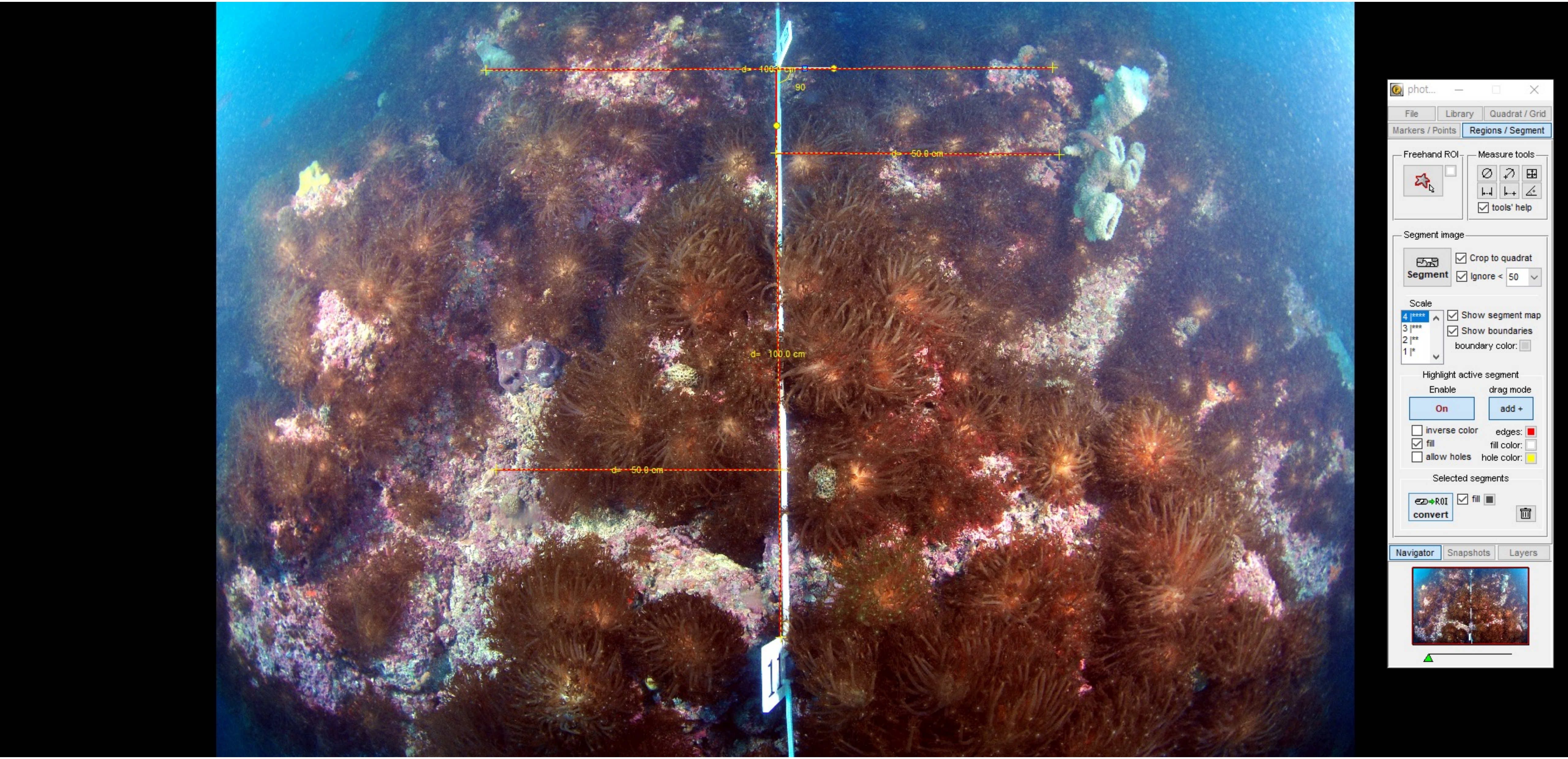

3. Quadrat detection (1 m x 1 m, $1 m^2$ virtual photo quadrat)

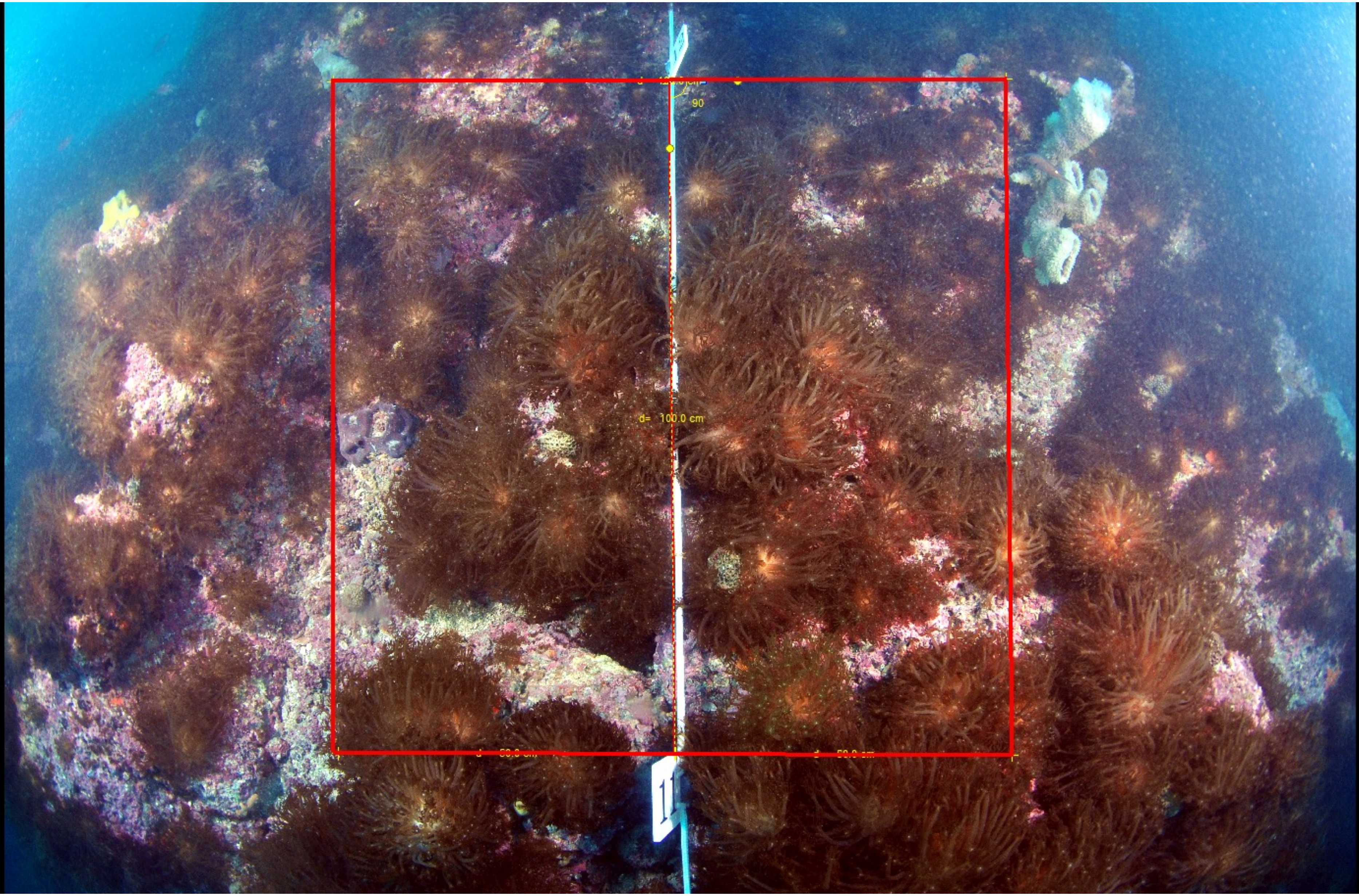

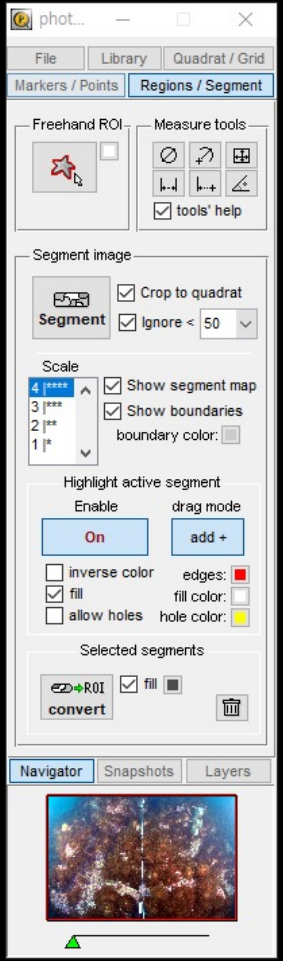

Supplement: S1 File — (PDF) [file pone.0275244.s001.pdf]
