# Supplementary material for: Dominance of the scleractinian coral Alveopora japonica in the barren subtidal hard bottom of high-latitude Jeju Island off the south coast of Korea assessed by high-resolution underwater images
Source: PLoS One. 2022 Nov 4;17(11):e0275244. doi: 10.1371/journal.pone.0275244 (PMC9635743; doi:10.1371/journal.pone.0275244)
Supplement: S2 File — (DOCX) [file pone.0275244.s002.docx]

**Dominance of the scleractinian coral *Alveopora japonica* in the barren subtidal hard bottom of high-latitude Jeju Island off the south coast of Korea assessed by high-resolution underwater images**

Kyeong-Tae Lee^1,4^, Hye-Mi Lee^1^, Thatchaneshkanth Subramaniam^1^ Hyun-Sung Yang^4^, Sang-Rul Park^1^, Chang-Keun Kang^2^, Shashank Keshavmurthy^3*^, Kwang-Sik Choi^1*^

^1^Department of Marine Life Science (BC 21 PLUS) and Marine Science Institute, Jeju National University, 102 Jejudaehakno, Jeju 63243, Republic of Korea

^2^School of Earth Sciences and Environmental Engineering, Gwangju Institute of Science and Technology, Gwangju 61005, Republic of Korea

^3^Biodiversity Research Centre, Academia Sinica, Nangang, Taipei 115, Taiwan

^4^Jeju Marine Research Center, Korea Institute of Ocean Science & Technology (KIOST), Jeju 63349, Korea

**Supplementary Table and Figures**

**Table S1.** Average percent cover of benthic organisms estimated on 20-m line transects installed at depths of 5 m, 10 m, 15 m of 3 survey sites (SH: Shinheung; BC: Bukchon; SS: Seongsan).

| **Species** | **Average percent cover (%)** | | | | | | | | |
| --- | --- | --- | --- | --- | --- | --- | --- | --- | --- |
|  | **SH** | | | **BC** | | | **SS** | | |
|  | **5 m** | **10 m** | **15 m** | **5 m** | **10 m** | **15 m** | **5 m** | **10 m** | **15 m** |
| *Callyspongia* sp. |  |  |  |  |  | 0.1 | 0.1 | 0.1 | 0.1 |
| *Halichondria* sp. |  |  | 0.1 |  |  | 0.2 |  |  | 0.1 |
| *Haliclona* sp. |  | 0.1 | 0.4 |  |  | 0.1 |  |  |  |
| *Hymeniacidon* sp. | 0.1 | 1.1 | 0.7 | 0.5 | 0.2 | 0.4 |  |  |  |
| *Petrosia* sp. |  | 0.2 |  |  |  |  |  |  |  |
| *Spirastrella* sp. | 0.1 | 0.3 | 2.2 |  |  | 0.6 |  | 0.2 |  |
| *Dendronephthya* sp. | 1.0 | 0.2 | 4.2 |  |  |  |  |  |  |
| *Heteractis* sp. | 0.1 | 0.1 |  |  | 0.2 |  |  |  |  |
| *A. japonica* | 10.0 | 0.8 | 0.2 | 0.4 | 45.9 | 72.8 | 0.1 |  | 15.3 |
| *Cardita* sp. |  |  |  |  |  | 0.1 |  |  |  |
| *Turbo* sp. | 0.1 | 0.2 |  | 0.1 |  |  | 0.1 |  |  |
| Unidentified annelid |  | 0.1 |  |  |  |  |  |  |  |
| *Adeonella* sp. |  |  | 0.1 |  |  |  |  |  |  |
| *Comatula* sp. |  | 0.1 |  |  |  |  |  |  |  |
| *Certonardoa* sp. | 0.1 |  | 0.2 |  | 0.1 |  |  | 0.1 |  |
| *Heliocidaris* sp. | 0.2 | 0.1 |  | 0.3 |  |  | 0.1 |  |  |
| *Herdmania* sp. |  | 0.1 | 0.1 |  |  |  |  |  |  |
| *Lissoclinum* sp. |  |  | 0.1 |  |  | 0.1 |  |  |  |
| *Cladophora* sp. | 0.1 | 0.5 | 0.1 | 0.1 | 0.2 |  |  | 6.5 | 0.7 |
| *Codium* sp. | 0.1 |  | 0.8 | 0.1 | 4.5 |  |  |  | 0.1 |
| *Colpomenia* sp. |  |  |  |  |  |  | 0.1 |  |  |
| *E. cava* | 0.1 | 0.1 | 0.3 | 0.4 |  |  | 20.2 | 3.2 | 3.1 |
| *Padina* sp. |  |  |  |  |  |  | 0.2 |  |  |
| *Sargassum* spp. |  |  |  | 1.4 |  |  | 24.3 | 0.3 | 1.0 |
| *Champia* sp. | 0.9 | 0.3 | 0.1 | 0.1 |  |  | 0.5 |  |  |
| *Gelidium* sp. | 0.1 | 0.3 |  |  |  |  |  | 0.8 |  |
| *Grateloupia* sp. |  |  | 0.1 |  |  |  | 0.5 | 4.4 | 4.2 |
| *Martensia* sp. | 0.6 | 0.4 |  | 4.1 | 0.1 |  |  |  |  |
| *Peyssonnelia* sp. |  |  |  |  |  | 0.1 | 0.2 | 1.9 | 8.0 |
| *Plocamium* sp. | 0.7 | 0.1 |  | 0.1 | 0.1 |  | 3.1 | 32.0 | 16.5 |
| *Pterocladiella* sp. |  |  |  |  |  |  | 0.3 | 1.6 |  |
| Geniculate corallines | 16.9 | 21.5 | 24.3 | 20.8 | 4.8 | 0.4 | 36.9 | 25.3 | 4.9 |
| Non-geniculate corallines | 63.3 | 69.0 | 60.2 | 68.6 | 42.3 | 22.3 | 11.6 | 19.7 | 39.4 |


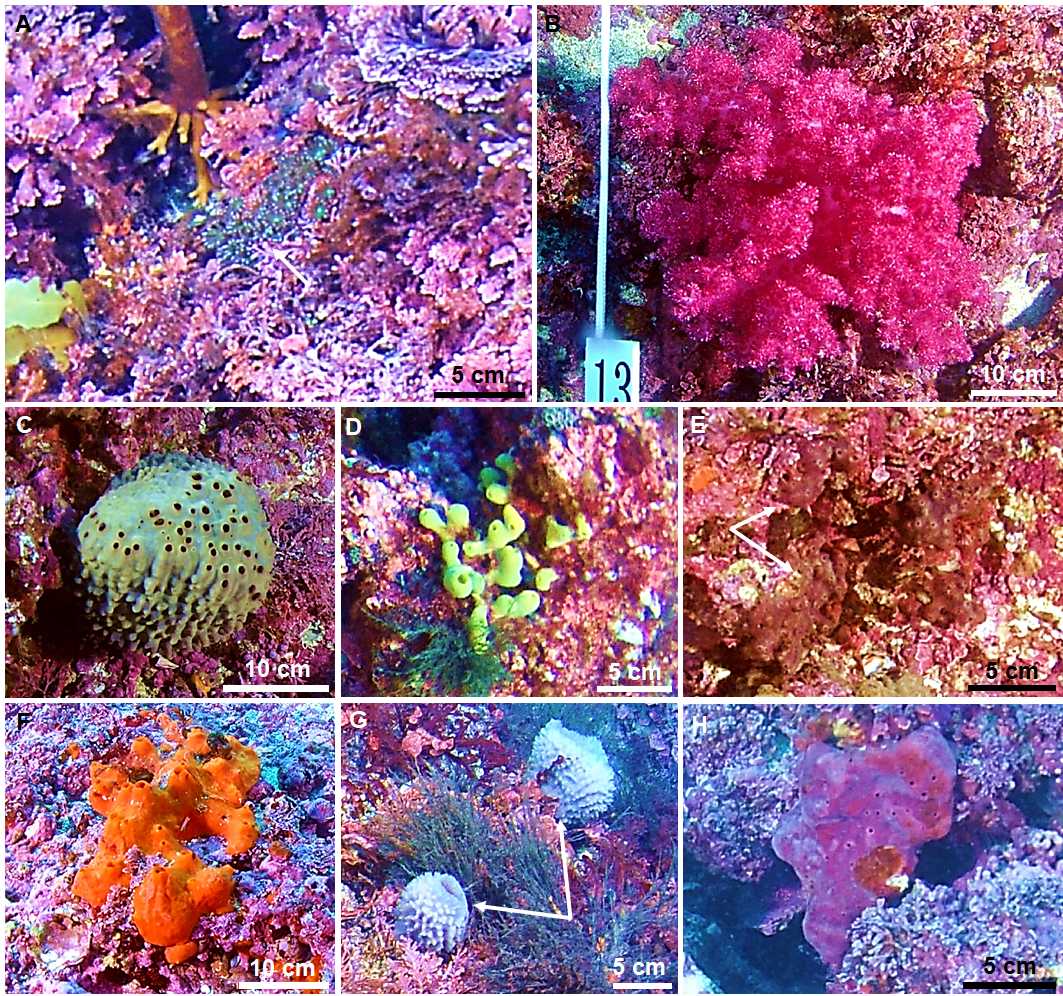


**Figure S2**. Hard coral, soft coral, sponge. Underwater images of benthic organisms identified from 20-m line transects installed at depths of 5 m, 10 m, 15 m of 3 survey sites (SH: Shinheung; BC: Bukchon; SS: Seongsan). (A) hard coral (*A. japonica*) SS, 5 m; (B) soft coral (*Dendronephthya* sp.) SH, 15 m. The benthic substrate covered with sponges including massive or encrusting sponges and branching sponges (C) *Spirastrella* sp. SH, 15 m; (D) *Halichondria* sp. SS, 15 m; (E) *Haliclona* sp. SH, 15 m; (F) *Hymeniacidon* sp. BC, 5 m; (G) *Callyspongia* sp. SS, 10 m; (H) *Petrosia* sp. SH, 10 m


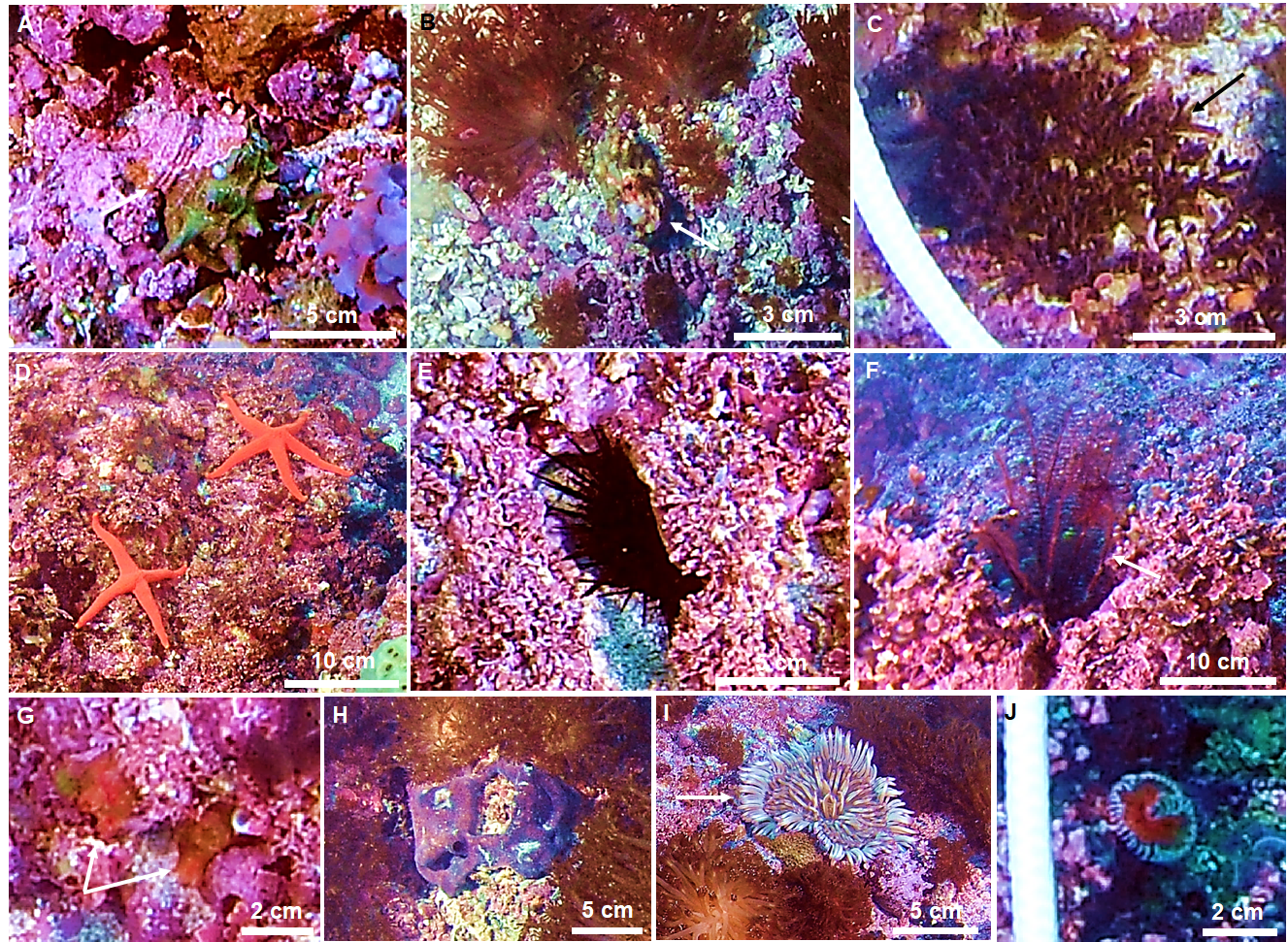


**Figure S3**. Mollusk, bryozoan, echinoderm, ascidian, anemone, annelid. Underwater images of benthic organisms identified from 20-m line transects installed at depths of 5 m, 10 m, 15 m of 3 survey sites (SH: Shinheung; BC: Bukchon; SS: Seongsan). (A) gastropod (*Turbo* sp.) partially encrusted with non-geniculate corallines SH, 5 m; (B) bivalve (*Cardita* sp.) BC, 15 m. (C) bryozoan (*Adeonella* sp.) SH, 15 m; (D) asteroid (*Certonardoa* sp.) SH, 15 m; (E) echinoid (*Heliocidaris* sp.) SH, 5 m; (F) crinoid (*Comatula* sp.) SH, 10 m; (G) individual ascidian (*Herdmania* sp.) SH, 5 m; (H) colonial ascidian (*Lissoclinum* sp*.*) BC, 15 m; (I) anemone (*Heteractis* sp.) BC, 10 m; (J) Unidentified annelid SH, 10 m.


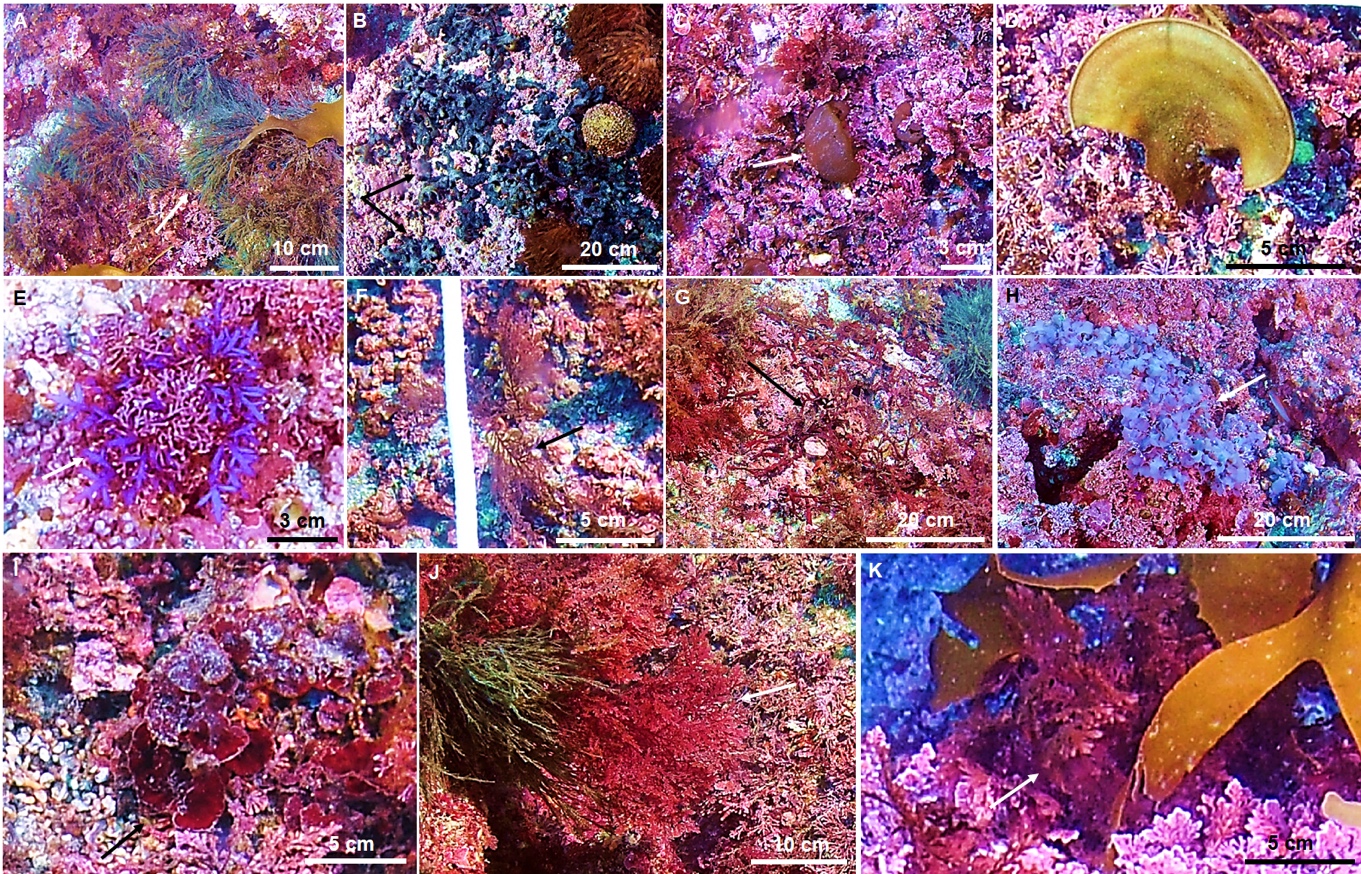


**Figure S4**. Chlorophyte, phaeophyte, rhodophyte**.** Underwater images of benthic organisms identified from 20-m line transects installed at depths of 5 m, 10 m, 15 m of 3 survey sites (SH: Shinheung; BC: Bukchon; SS: Seongsan). (A) *Cladophora* sp. SS, 10 m; (B) *Codium* sp. BC, 10 m; (C) *Colpomenia* sp. SS, 5 m; (D) *Padina* sp. SS, 5 m; (E) *Champia* sp. SH, 5 m; (F) *Gelidium* sp. SS, 10 m; (G) *Grateloupia* sp. SS, 10 m; (H) *Martensia* sp. SH, 10 m; (I) *Peyssonnelia* sp. SS, 15 m; (J) *Plocamium* sp. SS, 10 m; (K) *Pterocladiella* sp. SS, 5 m.
